# An *in vitro* transepithelial migration assay to evaluate the role of neutrophils in Respiratory Syncytial Virus (RSV) induced epithelial damage

**DOI:** 10.1038/s41598-018-25167-4

**Published:** 2018-04-30

**Authors:** Yu Deng, Jenny A. Herbert, Claire M. Smith, Rosalind L. Smyth

**Affiliations:** 1 Respiratory, Critical Care & Anaesthesia, Great Ormond Street Institute of Child Health, University College London (UCL), London, United Kingdom; 2 Department of Respiratory medical centre, Chongqing Key Laboratory of Child Infection and Immunity, Children’s Hospital of Chongqing Medical University, China International Science and Technology Cooperation base of Child development and Critical Disorders, Ministry of Education Key Laboratory of Child Development and Disorders, Chongqing, 400014 China

## Abstract

Large numbers of neutrophils migrate into the lungs of children with severe Respiratory Syncytial Virus (RSV) disease. It is unclear how these cells contribute to viral clearance and recovery from infection or whether they contribute to disease pathology. We have developed a novel *in vitro* model to study neutrophil migration through airway epithelial cells (AECs), the main cellular target of RSV infection. Our model reproduces a physiologically relevant cell polarity and directionality of neutrophil migration. Using this model, we found that RSV infected AECs induced rapid neutrophil transepithelial migration. We also detected increased AEC damage associated with RSV infection, with a further increase in epithelial cells shedding from the Transwell membrane following neutrophil migration. This was not observed in the mock infected controls. Neutrophils that migrated through the RSV infected AECs showed increased cell surface expression of CD11B and MPO compared to neutrophils that had not migrated. In conclusion, our *in vitro* co-culture assay can be used to identify critical mechanisms that mediate epithelial cell damage and promote inflammation in children with severe RSV disease.

## Introduction

Respiratory syncytial virus (RSV) is the leading cause of hospitalisation of infants in the developed world, causing bronchiolitis and severe lower respiratory tract disease in infants and young children^1^. There is currently no vaccine to prevent RSV infection and no specific anti-viral treatment.

The main target for RSV infection is the airway epithelial cells (AECs), specifically ciliated AECs^2,3^. Infection of AECs with RSV induces the production of pro-inflammatory mediators, including pro-inflammatory cytokines IL-6 and chemotactic cytokines CXCL8 (IL-8) and CXCL10 (IP-10), which lead to the recruitment of immune cells, including neutrophils^4–6^.

Neutrophils are an important part of innate host defence against RSV infection and are rapidly recruited to the airways to migrate from the basal sub-epithelial space across the epithelium to the airways following infection^7^. Massive pulmonary neutrophil infiltration is observed in paediatric patients with severe RSV induced bronchiolitis^8,9^. In one study, neutrophils accounted for 76% of infiltrated cells in the lower airways and 93% in the upper airways^8^. The mechanisms by which neutrophils migrate across lung epithelia are unclear and a better understanding of this process will likely provide new insights into novel treatment strategies.

A major obstacle to research in this area is a lack of appropriate models. Animal models of RSV infection often do not accurately reproduce the pathology of disease seen during human infection^10^. Very high viral doses are often required for any clinical symptoms to occur^10^. Furthermore, as severe RSV bronchiolitis is most prevalent in infants under 5 months of age^11^, there are ethical and technical limitations for clinical studies to collect appropriate samples.

A number of *in vivo* and *in vitro* methods have been used to study the responses of airway epithelium to RSV infection^2,10,12,13^. Cell culture systems provide important insights into host pathogen interactions. However, a fairly inexpensive *in vitro* model that enables the investigator to experimentally manipulate the viral infection, epithelial barrier, and/or neutrophil in a well-controlled, highly reproducible system is needed. This would be similar to that developed to model intestinal epithelial and lung epithelial responses to bacterial pathogens^14–21^, which use cells lines to model the epithelial monolayer. More recently a model has also been developed studying neutrophil migration during bacterial infection using primary airway epithelial cells^22^.

Here, we describe the development of a co-culture assay system to study the consequences of neutrophil trans-epithelial migration in the physiologically relevant basolateral to apical direction in response to RSV infection of the airways.

## Methods

### Participants

Peripheral blood was taken from healthy adult donors at UCL GOS Institute of Child Health. Written informed consent was obtained from all donors prior to their enrolment in the study. Study approval was obtained from the UCL Research Ethics Committee (4735/002). All methods were performed in accordance with the relevant guidelines and regulations.

### Epithelial cell culture

Human type-2 alveolar basal epithelial cells (A549) (ATCC CCL-185) were maintained in growth medium (RPMI Glutamax (Life Technologies) supplemented with 5% v/v newborn calf serum (NCS) (Life Technologies), 1mM L-glutamine (L-glut) (Life Technologies) and 1 × Penicillin/streptomycin (Life Technologies)). HEp-2 cells (ATCC CCL-23) were maintained in Opti-MEM media (Life Technologies) supplemented with 10% v/v NCS, 1mM L-glut and 1 × Penicillin streptomycin.

### Virus purification and quantification

Recombinant GFP tagged RSV A2 strain was kindly provided by Fix *et al*.^23^. Viral propagation was performed in HEp-2 cells (MOI 0.1) for 3–5 days in Opti-MEM with 1mM L-glut and 2% NCS. Infected cells were lysed in a sonicating water bath (Grant, XUBA1), followed by centrifugation at 1200 rpm for 5 minutes. Supernatant was collected and virus concentrated and purified as described previously^24^. Briefly, virus was purified by centrifugation through a polyethersulphone membrane with a pore size of 1000 000 Daltons MWCO (1000 kD) (Vivaspin-20, Vivascience, Gloucester, UK). Virus was collected in RPMI (Life Technologies), aliquoted and frozen at −80 °C. Viruses were quantified by plaque assay. Briefly, 2.5 × 10^4^ HEp-2 cells were seeded into a 96 well plate and cultured overnight in Opti-MEM plus supplements (as above) at 37 °C 5% CO_2_. The following day cells were washed in PBS and serial tenfold dilutions of viral stock added to triplicate wells (50 µl) for 2 hours at 37 °C 5% CO_2_. The inoculum was replaced with 200 µl of Opti-MEM for 24–48 hours at 37 °C and 5% CO_2_. Images of the whole well were captured using a Nikon Eclipse Ti-U microscope equipped with Hamamatsu ORCA 4.0 camera and fluorescence FITC filter. The number of viral plaques from each well were counted and pfu/ml was calculated by multiplying the plaques/well by dilution factor and x10.

### Neutrophil isolation and purification

Neutrophils were isolated from peripheral venous blood using a Percol density gradient as described previously^25^. A further purification step was performed using the EasySep™ Human Neutrophil Enrichment Kit (STEMCELL Technologies) as per the manufacturer’s instructions^26^. Neutrophils were resuspended in Hanks balanced salt solution (HBSS^−^) without calcium and magnesium (Life Technologies) and FACS analysis was performed using Anti-Human CD49d-APC (BioLegend, 304307) and Anti-Human CD66a/c/e AlexaFluor-488 (BioLegend, 342306) antibodies to confirm purity. Prior to migration, neutrophils were stained with a CellTrace Calcein Red-Orange cell stain (Thermo Fisher) as per the manufacturer’s instructions. Stained neutrophils were washed twice in HBSS^−^ then resuspended in HBSS plus calcium and magnesium (HBSS^+^). Unstained neutrophils were used if downstream analysis was to be performed (FACS/ microscopy), these were resuspended in HBSS^+^ prior to migration.

### RSV infection of AECs and transepithelial neutrophil migration

**Figure 1:**
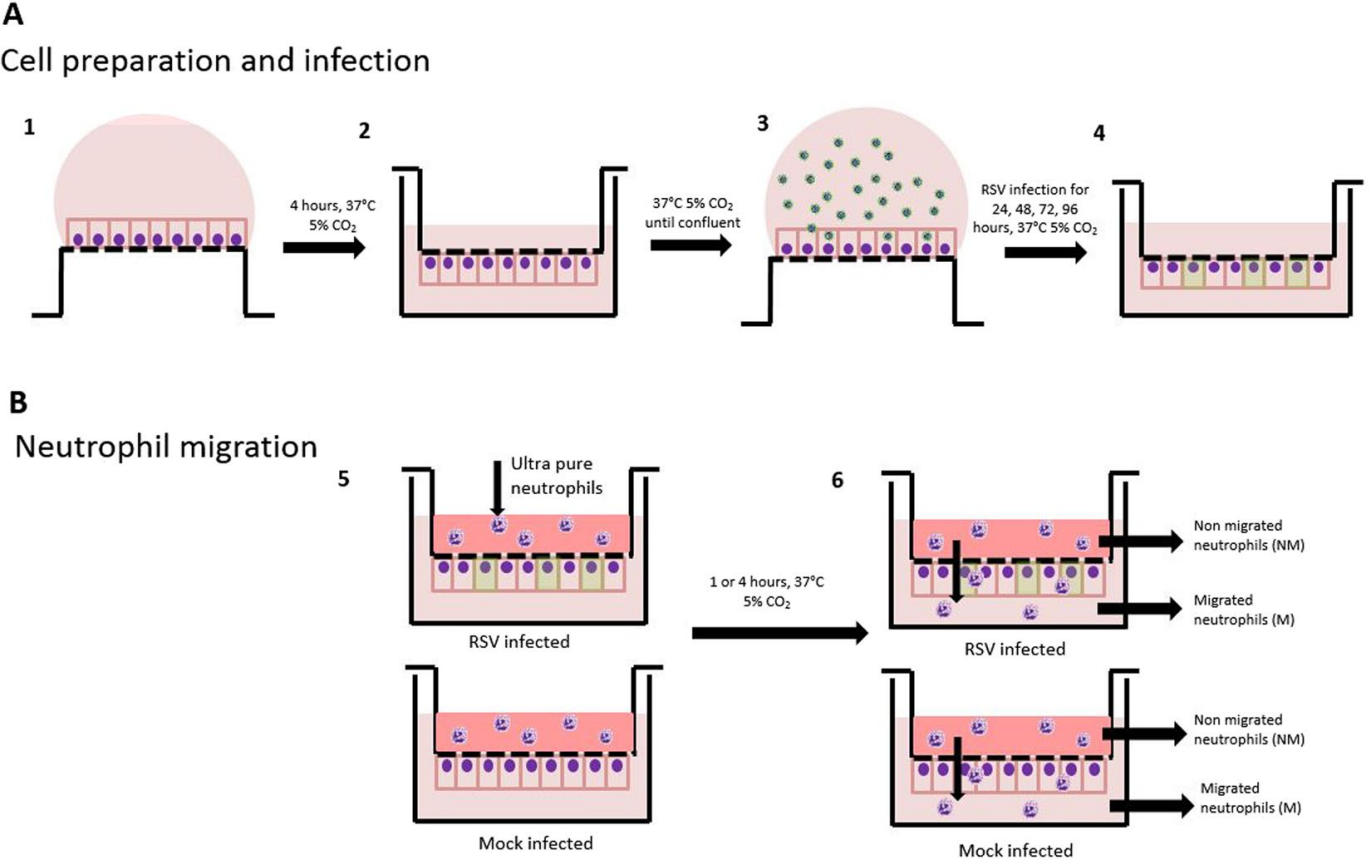
Schematic diagram of neutrophil migration model. Diagram of cell seeding, A549 infection **(A)** and neutrophil migration **(B)**. (1) A549 cells were seeded onto the underside of a transwell and allowed to attach for 4 hours. (2) Transwells were subsequently inverted and maintained in media to allow a confluent epithelial monolayer to develop for 72 hours. (3) Transwell were inverted and infected with GFP RSV or mock infected for 2 hours. (4) Transwell*s were inverted* and maintained in media and infection allowed to progress for 24, 48, 72 or 96 hours. (5) Ultrapure neutrophils were added to the basolateral side of the transwell, and were allowed to migrate for 1 or 4 hours. Underneath the transwell was HBSS+ or RSV infection media. (6) Post migration basolateral and apical neutrophils were collected and downstream analysis performed.

A schematic diagram of our neutrophil migration assay is shown in Fig. 1. A549 cells (1 × 10^5^) were seeded on the underside of a 24 well PET transwell (8 µm pore membrane) for 4 hours at 37 °C, 5% CO_2._ Transwells were then inverted and maintained in growth medium (as above) until confluent (Figure S1A). Cells were then rinsed with PBS and infected with recombinant GFP tagged RSV A2 strain^23^ at MOI 1, or mock infected (RPMI only) for 2 hours at 37 °C, 5% CO_2_. The inoculum was then removed and Transwells were rinsed with RPMI then placed into RPMI + 1% FBS for up to 96 hours.

Transwells were placed in a new ultra-low binding 24 well plate (Corning, Costar). 350 µl of HBSS^+^ or infection media (media collected from AECs) was added to the bottom (apical side) and 5 × 10^5^ neutrophils in HBSS^+^ were added to the top (basolateral side) of each Transwell. As a positive control, 100nM N-Formylmethionine-leucyl-phenylalanine (fMLP, Sigma) in 350 µl of HBSS+ was added to the apical side of the cells. All neutrophils were allowed to migrate for 1 and 4 hours. Neutrophils were then collected for downstream analysis.

### Quantification of neutrophil numbers

Non-migrated and migrated neutrophils were placed in triplicate into a 96 well flat bottom ultra-low binding plate (Corning, Costar and were quantified using a fluorescence plate reader (ex 584 nm/em 612 nm) (FLUOstar OPTIMA, BMG). Total neutrophils numbers were extrapolated from a standard curve of known numbers of fluorescent neutrophils, starting at 2.5 × 10^5^ in two fold serial dilutions. At least three biological replicas were performed for each group. Differences between Mock and RSV infected group were analysed using a non-parametric Mann-Whitney t-test (GraphPad Prism v5.0).

### Quantification of viral replication

Fluorescence microscopy was used to visualise GFP RSV infected cells. Cells were fixed with 1% (v/v) paraformaldehyde (PFA) for 10 minutes at room temperature, washed twice in PBS, followed by incubation with 50 µl of Hoechst stain (ThermoFisher) for 10 minutes at room temperature in the dark and washed once with sterile water. Membranes were removed from the transwell using a scalpel and mounted onto a glass microscope slide. Images were taken using a fluorescence microscope (Nikon Eclipse Ti-U) equipped with x20 objective and a Hamamatsu ORCA 4.0 camera and fluorescence DAPI and FITC filter.

RNA was extracted from A549 cells using an RNeasy RNA extraction kit (Qiagen) as per the manufacturer’s instructions. Cells were lysed directly on the membrane using RLT buffer. A DNase step was performed after RNA isolation using the TURBO DNA-free (Ambion) as per the manufacturer’s instruction to remove any contaminating DNA. RNA was quantified using the Nanodrop 1000 (ThermoFisher) and quality assessed using the Bioanalyser (Agilent). cDNA was synthesised using the High-Capacity RNA-to-cDNA kit (Applied Biosystems) as per the manufacturers instruction. A starting concentration of 0.5 µg total RNA was used in a final reaction volume of 20 µl. A no reverse transcriptase control was also performed to control for remaining contaminating DNA in the RNA preparation. Quantitative RT-PCR was performed using TaqMan Universal Master Mix II, with UNG (Applied Biosystems). A total reaction volume of 20 µl was used with 1 µl of cDNA per reaction. Primers and probes were used as previously described^27^. Primers were used at a final concentration of 10 mM and probe used at a final concentration of 2pM. Samples were run on an AB Biosystems step one plus RT-PCR machine. Reaction conditions- 1 cycle 50 °C for 2 minutes, 1 cycle 95 °C for 10 minutes, 40 cycles 95 °C for 15 seconds followed by 60 °C for 1 minute. A plasmid containing the N protein sequence^28^ was used to quantify the N protein copy number within cells. RSV load was extrapolated from a standard curve of known N protein copies. Differences in viral load at RSV infection time points was analysed using a paired t-test (GraphPad Prism v5.0).

### Quantifying cell damage (epithelial integrity)

Cell damage was quantified by Transepithelial electrical resistance (TEER), Red-dextran leakage, lactate dehydrogenase (LDH) release and by counting the number of cells that remained attached to the membrane (fluorescence microscopy). TEER was measured using an EVOM voltohmmeter (World Precision Instruments, Sarasota, FL, USA) after addition of 0.5 ml of culture medium to the apical surface. The concentration of the cytoplasmic enzyme LDH in the apical supernatants was determined using the Pierce LDH Cytotoxicity Assay Kit (Thermo fisher) as per the manufacturer’s instructions. Absorbance (490 nm) was measured using a plate reader (FLUOstar OPTIMA, BMG). Each sample was run in duplicate or triplicate depending on the amount of sample. Differences between Mock and RSV infected group were analysed by unpaired t-test.

Permeability of A549 monolayer was determined by flux of Texas red-conjugated 3-kDa dextran (Invitrogen, Camarillo, CA) from the apical to the basal chamber. In brief, after neutrophils had been allowed to migrate for 1 or 4 hours, transwell inserts were moved to a fresh 24-well plate containing 450 ul of pre-warmed HBSS in the outer chamber. Texas red-conjugated 3-kDa dextran solutions (5 mg/mL in HBBS) were added to the basolateral side of the epithelial cell layer and incubated at 37 °C. After 2 h incubation, inserts were removed and three 100 ul aliquots were transferred to a 96-well plate. The concentration of Texas red-conjugated 3-kDa dextran was calculated from the amount of fluorescence emission at 610 nm (excitation at 587 nm) using a titration curve of known concentration of the same tracers. The results were expressed as the percentage of mock group.

The number of epithelial cells and neutrophils remaining on the PET Transwell membrane post RSV infection and post neutrophil migration was determined using fluorescence microscopy. Membranes were fixed with 1% (v/v) paraformaldehyde (PFA) for 10 minutes at room temperature, washed twice in PBS and incubated with 50 µl of Hoechst stain (ThermoFisher) for 10 minutes at room temperature in the dark. Membranes were washed once with sterile water, removed from the Transwell using a scalpel and mounted onto a glass microscope slide. At least five Images were acquired from each slide using a confocal microscope (Zeiss LSM710) under a x40 objective. Areas were chosen where no cell clumps were observed as these prevented individual nuclei from being counted. The number of DAPI stained epithelial and neutrophil nuclei in each image were counted using the ImageJ counting tool.

### Cytokine production

CXCL8 (IL-8) and IL-6 levels in apical supernatants were quantified using DuoSet ELISAs (R&D systems) as per the manufacturer’s instruction. Each sample was run in duplicate and at least three biological replicas were performed for each group. Differences between Mock and RSV infected group were analysed by unpaired t-test.

### Neutrophil CD11B and MPO expression

The amount of neutrophil activation was determined by measuring the cell surface protein expression levels of CD11B and myeloperoxidase (MPO). Neutrophils that were not used for migration experiments were used as a negative control. All neutrophils were centrifuged at 1400 rpm, 5 mins and washed once in 500 µl FACS buffer (PBS (Ca^2+^ Mg^2+^ free), 0.5% Bovine serum albumin (BSA), 2.5 mM EDTA). The cell pellet was then resuspended in 50 µl (1/50 dilution in FACS buffer) of TruStain FcX blocker antibody (BioLegend) and incubated at 4 °C for 10 minutes, then washed In FACS buffer as noted above. Cells were resuspended in 50 µl FACS buffer plus 1/250 dilution PE anti-Human CD11b conjugate (50-0118-T100, Insight Biotechnology) and 1/50 dilution Anti-MPO-APC human antibody (130-107-177, clone: REA491, Miltenyl Biotec) and incubated at 4 °C for 20 minutes in the dark. Unstained and single antibody controls were performed and confirmed no cross reactivity. Cells were washed once in FACS buffer, resuspended in 1% PFA and stored at 4 °C. Directly prior to running, samples were centrifuged (1400 rpm) and resuspended in FACS buffer.

Samples were analysed using a Beckton Dickenson LSR II flow cytometer and FlowJo v10.0 FACS analysis software. Neutrophils were first identified as being CD11b positive (PE+). Using this population the geometric mean fluorescence intensity of this population for PE and APC were calculated. Statistical analysis was preformed using a paired t-test (GraphPad Prism v5.0).

## Results

### Characterisation of *in vitro* transepithelial neutrophil migration model

**Figure 2:**
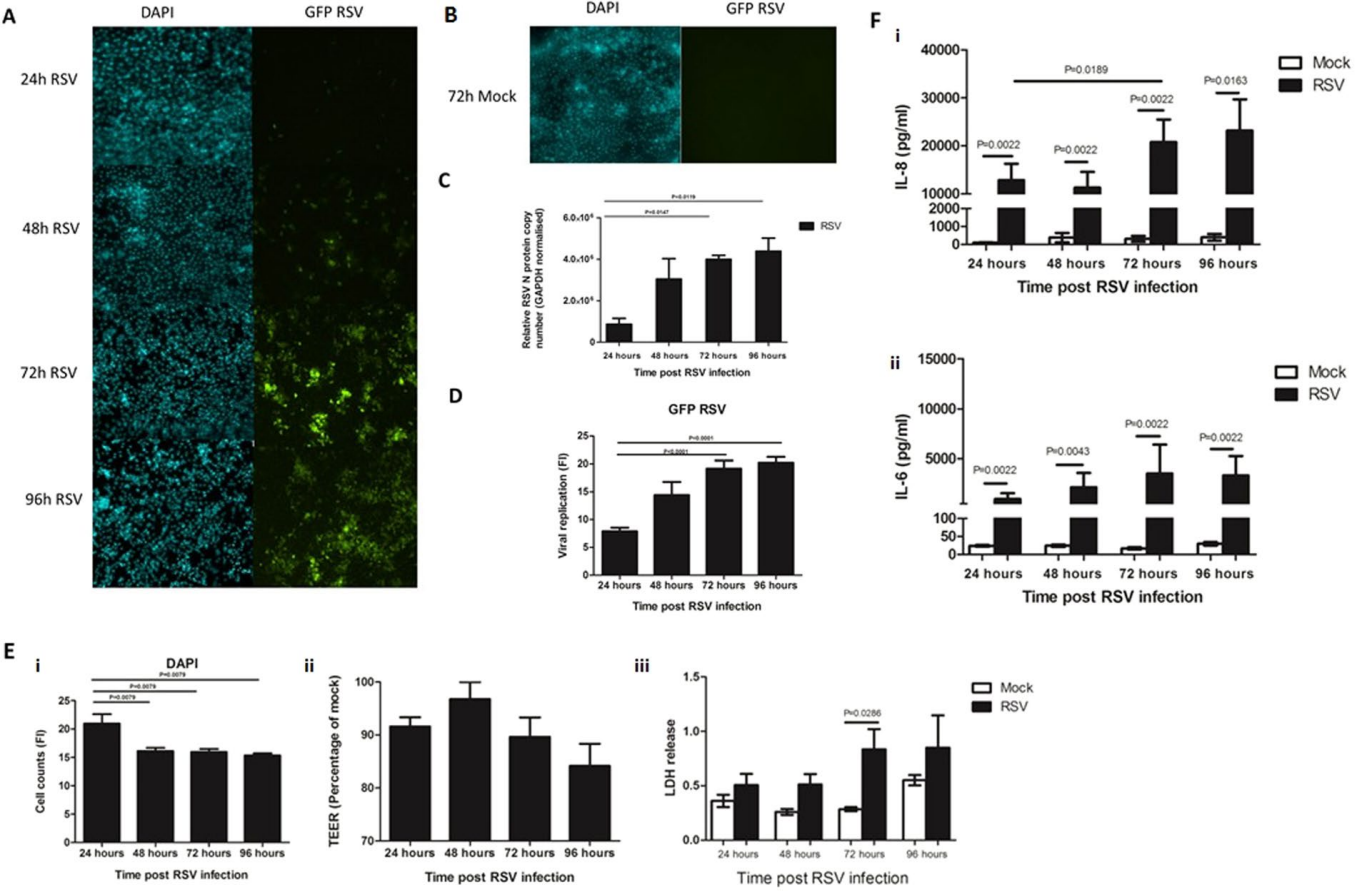
Characterisation of our RSV infection model. **(A)** Representative microscopy images of RSV infected A549 epithelial monolayer with green fluorescence (GFP) and DAPI fluorescence over time. **(B)** Representative microscopy images of mock infected A549 monolayer with GFP and DAPI fluorescence at 72 h post infection. No GFP fluorescence is observed in these controls. **(C)** Quantitative analysis of the images shown in Fig. 2A. GFP and DAPI fluorescence intensity was quantified using ImageJ, at least 4 images were used for each time point. Bars show mean ± SEM. Statistical significance is shown. **(D)** Quantitative RT-PCR of RSV viral load in A549 cells as measured by N protein copy number. Viral load was measure in A549 cells infected with GFP RSV over time. Bars show mean ± SEM of n = 3 biological repeats. Statistical significance is shown. **(E)** The damage caused to the A549 epithelium following RSV infection for 24, 48, 72 and 96 hours as determined by (i) number of epithelial nuclei/cell remaining attached to the membrane based on DAPI staining/0.045mm^2^ (ii) TEER as measured using a voltohmmeter, data is represented as the percentage relative to the mock infected control (iii) Lactate dehydrogenase (LDH) release as measured in supernatant of AECs post RSV infection. Statistical significance is shown. Bars show mean ± SEM of at least n = 3 biological repeats. **(F)** CXCL8 (IL-8) and IL-6 concentrations in supernatants post RSV and mock infection of A549 cells as measured by ELISA. Bars show mean ± SEM of n = 3 biological repeats. Statistical significance is shown. For all other comparisons P > 0.05.

In order to compare this infection model to those previously reported, initial experiments were performed to assess viral replication, epithelial monolayer integrity and inflammatory response. As predicted, we observed an increase (P < 0.05) in fluorescence in GFP-RSV infected transwells over time (Fig. 2A and C) from a mean ± SEM of 7.8. ± 0.7 arbitrary units at 24 hours post infection to 19.2 ± 1.4 at 72 hours and 20.3 ± 1.0 at 96 hour post infection (Fig. 2C). No GFP fluorescence was detected in mock infected transwells at all time points tested (Fig. 2B). The increase in GFP fluorescence correlated with an increase in RSV N-protein copy number (Fig. 2D). These data showed that viral load significantly (P < 0.05) increased from 24 to 72 h and 96 h post RSV infection. Previous data generated in our lab has also indicated viral load peaks at 72 hours post RSV infection^29^.

RSV infection also resulted in significant damage to the A549 cell monolayer with a significant (P < 0.05) reduction in the number of epithelial cells that remained attached to the Transwell compared to the mock infected control (Fig. 2E). We observed a decrease in number of DAPI stained epithelial cell nuclei between 24 (21.0 ± 1.7) and 48 hours (16.1 ± 0.6), 72 hours (16.0 ± 0.5) and 96 hours (15.4 ± 0.4) post RSV infection, suggesting fewer cells remain on the membrane after 24 hours. We also detected a significant (P < 0.05) increase in LDH release (2.9 fold increase) after 72 hours infection. The LDH release in the mock controls remained stable at 24, 48, and 72 hours post infection. RSV infection of AECs has also been shown to decrease TEER of epithelial monolayers^30^, however we did not detect a significant change in TEER between the time points (Fig. 2E).

A characteristic of RSV infection is the production of large amounts of chemokines and cytokines^5,6,29^. We also found that the amount of CXCL8 (IL-8) and IL-6 in supernatants significantly (P < 0.05) increased following RSV infection compared to the mock controls (Fig. 2F). We also found that the amount of IL-8 significantly (P < 0.05) increased between 24 hours (12.8 ± 3.4 ng/ml) and 72 hour (20.8 ± 4.7 ng/ml) post RSV infection.

### RSV infection increases neutrophil transepithelial migration

**Figure 3:**
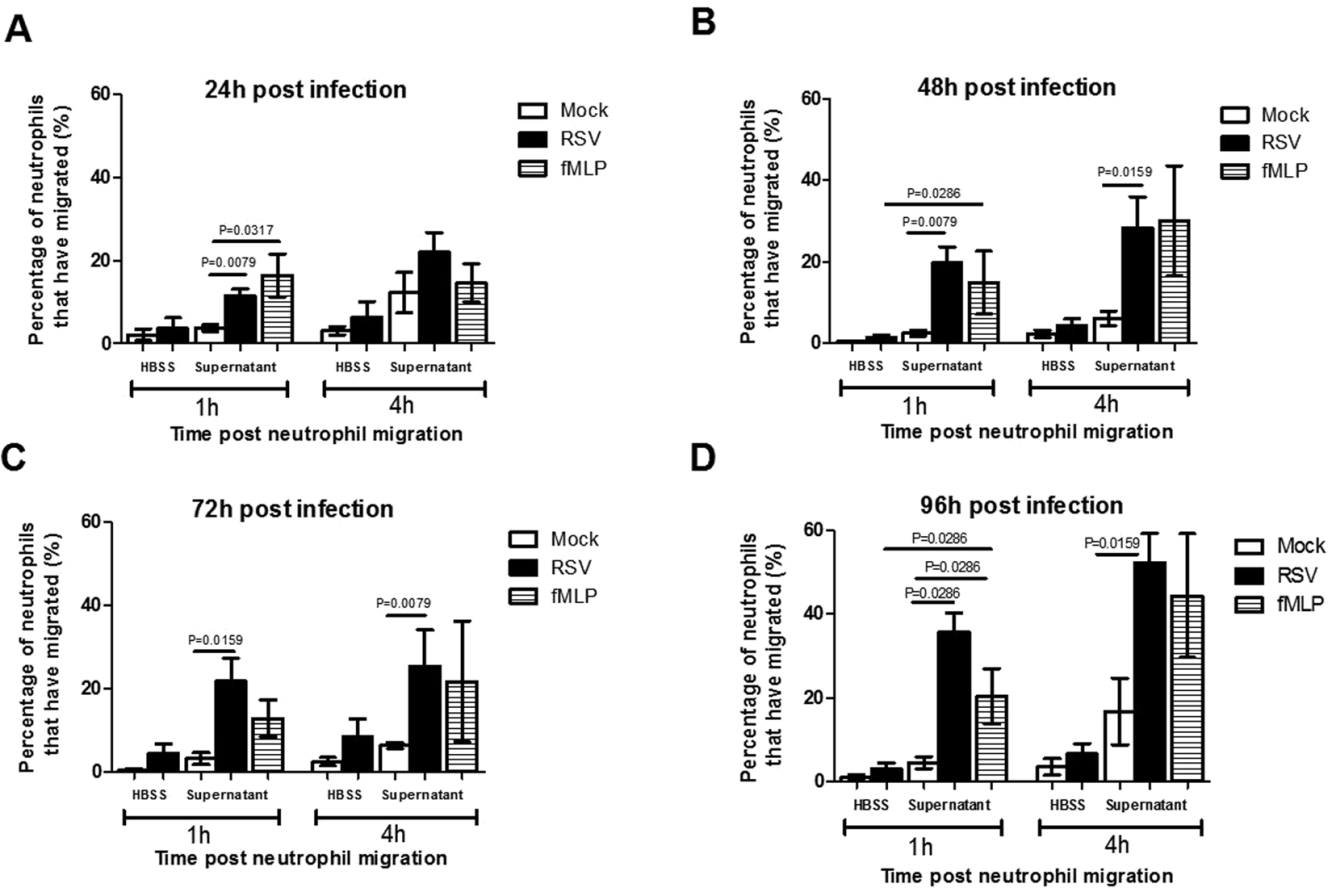
RSV increases the numbers of neutrophils that migrate through infected A549 monolayers The percentage of neutrophils that migrated through mock and RSV infected A549 monolayers after **(A)** 24 hours, **(B)** 48 h, **(C)** 72 h or **(D)** 96 hours post-infection was quantified. Neutrophils were exposed to infected A549 epithelial cells submerged in fresh HBSS^+^ or the conditioned supernatant produced from the same cells prior to migration (RSV and mock infected). The neutrophil chemoattractant N-Formylmethionine-leucyl-phenylalanine (fMLP) was used as a positive control. Bars show mean ± SEM of n = 3/4 biological repeats. Statistical significance is shown. For all other comparisons P > 0.05.

The number of neutrophils that migrated to the apical side of AECs after 1 hour significantly (P < 0.05) increased with RSV infection (11.5 ± 1.7%) compared to mock infected control (3.8 ± 0.8%) (Fig. 3A). This was found at all time points post-RSV-infection (24, 48, 72 and 96 hours) compared to their respective mock control (Fig. 3A–D) when conditioned supernatant (collected from cells prior to migration) was used. There was no significant change in the numbers of migrated neutrophils between the RSV and mock infected AECs when HBSS+ was used. This indicates that the presence of factors secreted into the supernatant during RSV infection are important for neutrophil migration.

RSV infection increased neutrophil migration in a time dependent manner (Table 1 and Figure S3/S4). After 1 hour a mean of 5.7 × 10^4^ neutrophils/well had migrated through RSV infected epithelial cells at 24 hours, 9.9 × 10^4^ at 48 hours, 1.1 × 10^5^ at 72 hours and 1.8 × 10^5^ at 96 hours post RSV infection, but the difference between these time points was not significant. The number of migrated neutrophils in the respective mock controls remained constant between 1.9 × 10^4^ and 2.3 × 10^4^ at 24 and 96 hours, respectively. After 4 hours the number of migrated neutrophils was slightly increased (P > 0.05) with counts of 1.1 × 10^5^, 1.4 × 10^5^, 1.3 × 10^5^ and 2.6 × 10^5^ neutrophils/well at 24, 48, 72 and 96 hours post RSV-infection respectively. As before, the number of migrated neutrophils in the mock controls remained constant between 6.1 × 10^4^ and 8.4 × 10^4^ at 24 and 96 hours, respectively (Fig. 3).

**Table 1:** Number of migrated neutrophils.

Time P.I	Time P.N	Mock infected		RSV infected	
		HBSS++	Conditioned Media	HBSS++	Conditioned Media
24 H	1 h	3655 ± 1573	19231 ± 10628	17947 ± 10605	51749 ± 15726*
	4 h	21341 ± 30410	37403 ± 14484	39491 ± 42521	92448 ± 42912
48 H	1 h	2796 ± 1721	12442 ± 10079	14608 ± 9045	96282 ± 50232*
	4 h	8748 ± 6668	102918 ± 134198	25209 ± 17761	156386 ± 90975
72 H	1 h	2966 ± 1841	8650 ± 3801	15544 ± 8985	102708 ± 68672*
	4 h	28437 ± 23953	31041 ± 9711	54116 ± 42995	136580 ± 109902
96 H	1 h	6554 ± 6779	20831 ± 16748	23480 ± 18864	213950 ± 89125*
	4 h	18156 ± 17044	97870 ± 95784	37029 ± 28396	287374 ± 60161*

P.I indicates post-infection. P.N. indicates post-neutrophil migration. Mean and SD shown (n = 4), boldface and * indicates significant increase in migrated neutrophils compared to respective Mock infected control.

### Migrated neutrophils express higher levels of CD11b and MPO in response to RSV infection compared to non-migrated neutrophils

**Figure 4:**
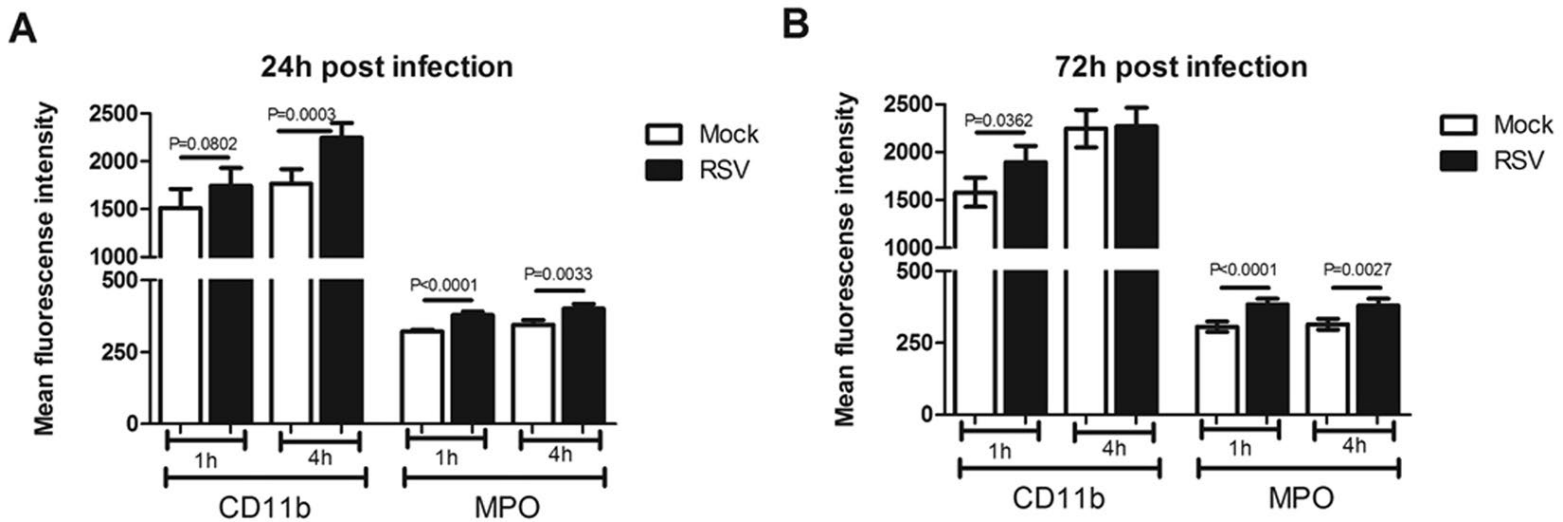
Neutrophil migration through RSV infected monolayers show increased cell surface expression of CD11B and MPO. Neutrophils that migrated through RSV infected epithelial cells infected for **(A)** 24 hours or **(B)** 72 hours showed higher cell surface expression of CD11B and MPO compared to those that migrated through mock-infected epithelial cells. Neutrophils were collected from the apical compartments and the percentage stained for cell surface expression of CD11B (PE) and MPO (APC) were determined by flow cytometry. Neutrophils were gated on initially using a PE positive gate. Using this population the geometric mean fluorescence intensity of PE and APC fluorescence was calculated. Neutrophils from the same donor were compared. Bars show mean ± SEM of n = 4 biological repeats. Statistical significance is shown. For all other comparisons P > 0.05.

Expression of CD11B and MPO was significantly (P < 0.05) increased on neutrophils that had migrated through RSV infected AECS after 1 hour compared to neutrophils that migrated through the mock infected AECs (Fig. 4). This was observed at 24 h and 72 h post-RSV infection (Fig. 4). CD11B has been used previously as a marker of neutrophil transmigration and MPO a marker of neutrophil activation^31^.

**Figure 5:**
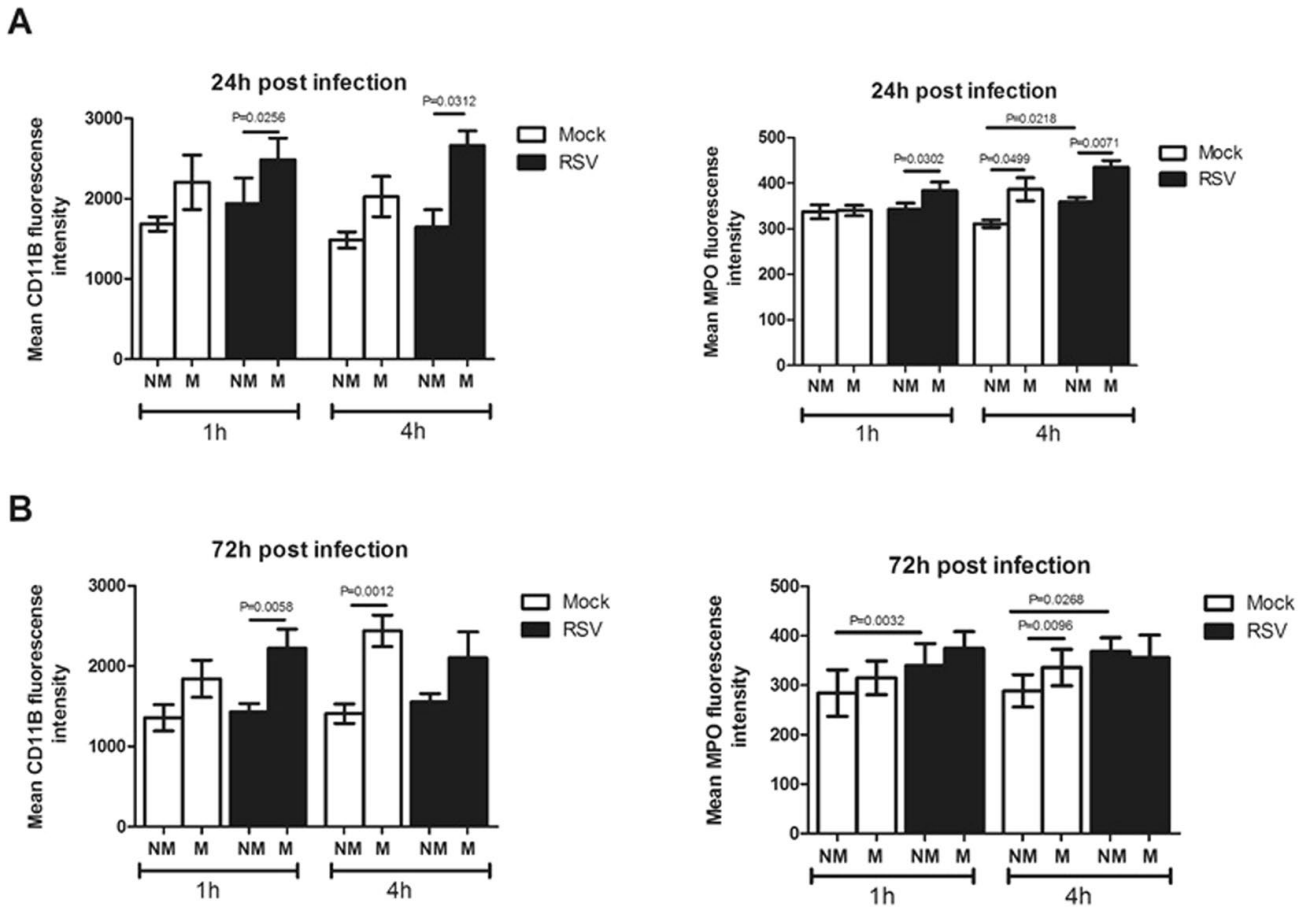
Migrated neutrophils show increased cell surface expression of CD11B and MPO compared to non-migrated neutrophils. Neutrophils that migrated (M) to the apical compartment through RSV infected epithelial cells infected for **(A)** 24 hours or **(B)** 72 hours showed higher cell surface expression of i) CD11B and ii) MPO compared to those non-migrated (NM) neutrophils that remain on the basolateral side. The percentage of migrated and non-migrated neutrophils expressing CD11B and MPO was calculated by staining for cell surface expression of CD11B (PE) and MPO (APC) were determined by flow cytometry. Neutrophils were gated on initially using a PE positive gate. Using this population the geometric mean fluorescence intensity of PE and APC fluorescence was calculated. Bars show mean ± SEM of n = 4 biological repeats. Statistical significance is shown. For all other comparisons P > 0.05.

We also found that expression of both CD11B and MPO was significantly (P < 0.05) increased on neutrophils that had migrated through epithelial cells infected with RSV for 24 hour compared to non-migrated neutrophils (Fig. 5A). We also found that expression of CD11B, but not MPO, was significantly (P < 0.05) increased in neutrophils which had migrated through AECs infected with RSV for 72 hour compared to non-migrated neutrophils after 1 hour (Fig. 5B).

### Neutrophil transepithelial migration enhances RSV infection induced epithelial monolayer disruption

**Figure 6:**
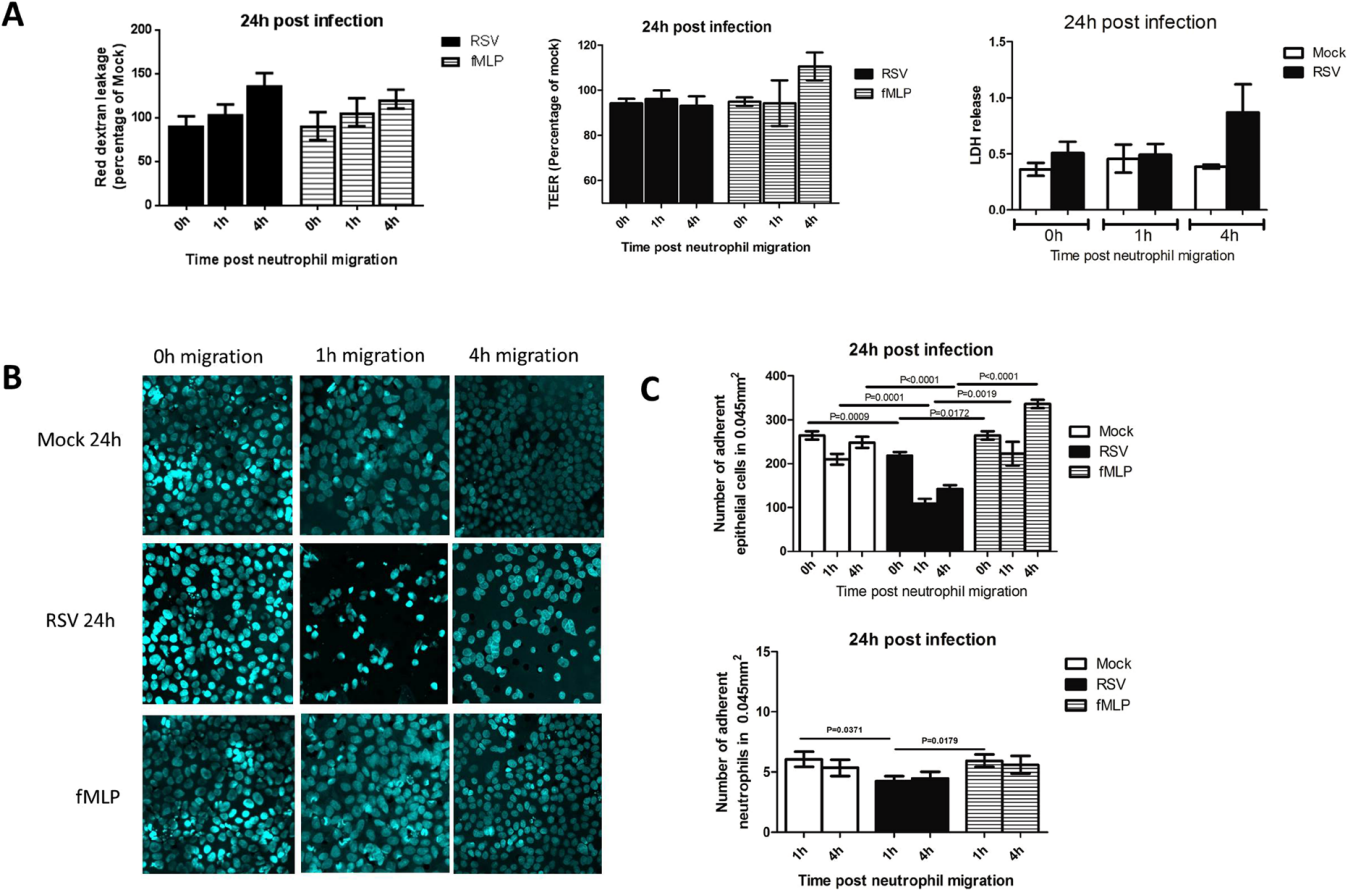
Damage caused by neutrophil migration through epithelial monolayers infected with RSV for 24 hours. **(A)** The damage caused to the A549 epithelium following neutrophil migration through cells infected with mock or RSV infection for 24 hours as determined by (i) Red-dextran leakage from basal-apical compartments, data represented as the percentage relative to the mock infected control n = 4 (ii) TEER as measured using a voltohmmeter, data is represented as the percentage relative to the mock infected control n = 9 (iii) Lactate dehydrogenase (LDH) release as measured in apical supernatant of AECs post neutrophil migration. The neutrophil chemoattractant N-Formylmethionine-leucyl-phenylalanine (fMLP) was used as a positive control Bars show mean ± SEM of at least n = 3 biological repeats. **(B)** Representative microscopy images of A549 epithelial monolayers infected with RSV for 24 hours stained with a nuclei stain (Hoechst) following neutrophil migration after 1 or 4 hours. **(C)** Epithelial cell and neutrophil nuclei were counted in ImageJ and the average number of nuclei from all images was calculated. Bars show mean ± SEM of n = 5 biological repeats. Statistical significance is shown. For all other comparisons P > 0.05.

To determine whether neutrophil migration enhances the cytopathology of RSV-infection, we measured cell damage in relation to red-dextran leakage (from basolateral to apical compartments), TEER and lactate dehydrogenase (LDH) release into the apical supernatant. We did not detect any significant increase in red-dextran leakage or decrease in TEER following neutrophil migration at 24 hours post RSV infection (Fig. 6A). However, the numbers of epithelial cells present on membrane after neutrophil migration significantly decreased following 24 hours RSV-infection compared to the respective mock controls (Fig. 6B and C). We found that 24 hours post RSV infection, neutrophil migration led to a significant (P < 0.05) 48% reduction in the number of AECs present on the membrane compared to the mock control (Fig. 6C). The number of adherent neutrophils also significantly (P < 0.05) decreased with RSV-infection compared to the mock-control (Fig. 6C). These data suggests that the fluorescence microscopy assay (cell adherence) may be a more sensitive method for analyzing cell damage in our model compared to the TEER and LDH methods (Further TEER data is shown in Figure S1/S2).

**Figure 7:**
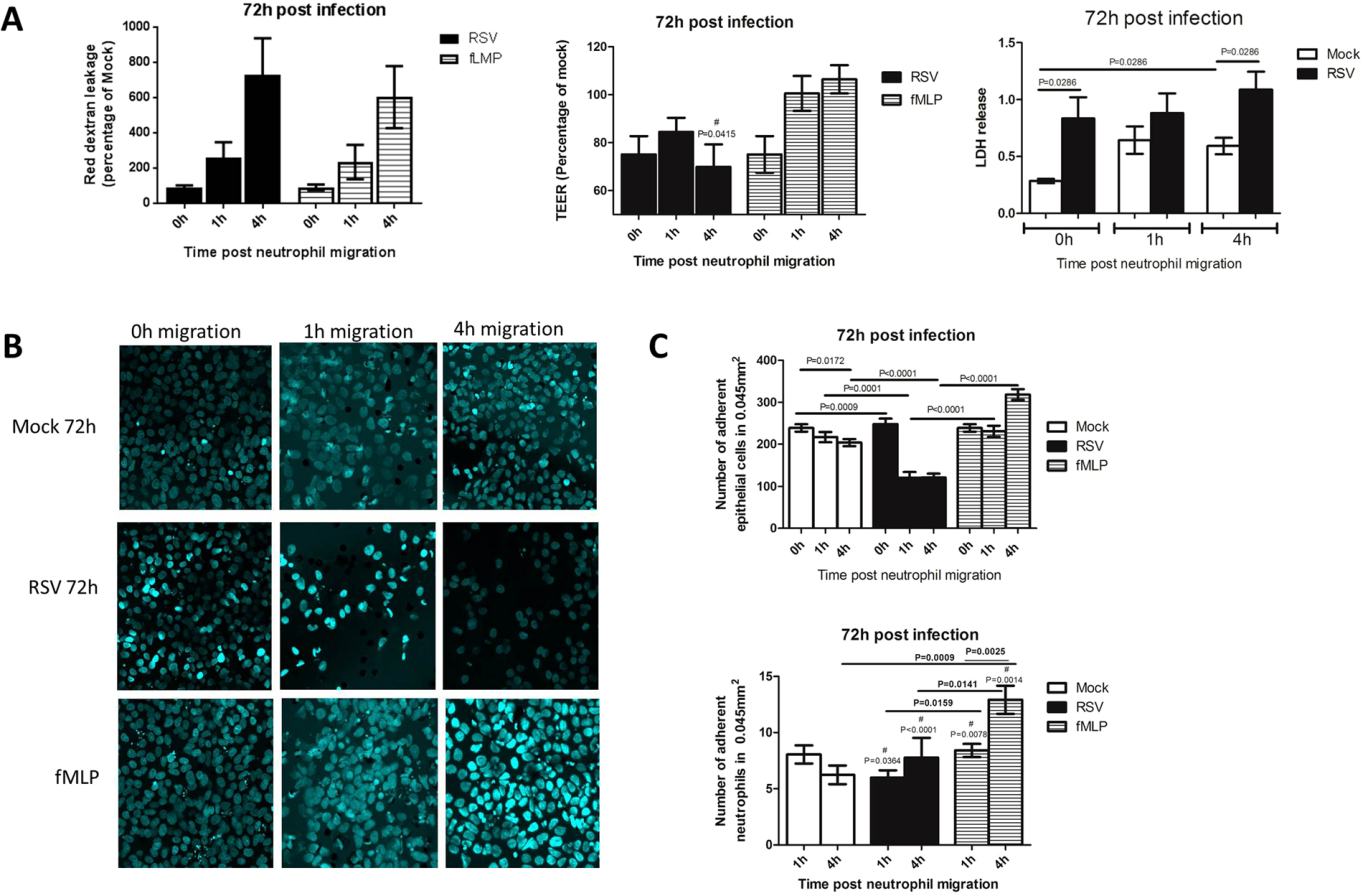
Neutrophil migration increases damage to epithelial monolayers infected with RSV for 72 hours. **(A)** The damage caused to the A549 epithelium following neutrophil migration through cells infected with mock or RSV infection for 72 hours as determined by i) Red-dextran leakage from basal-apical compartments, data represented as the percentage relative to the mock infected control n = 4 ii) TEER as measured using a voltohmmeter, data is represented as the percentage relative to the mock infected control n = 9 iii) Lactate dehydrogenase (LDH) release as measured in apical supernatant of AECs post neutrophil migration. The neutrophil chemoattractant N-Formylmethionine-leucyl-phenylalanine (fMLP) was used as a positive control. Bars show mean ± SEM of at least n = 3 biological repeats. **(B)** Representative microscopy images of A549 epithelial monolayers infected with RSV for 72 hours stained with a nuclei stain (Hoechst) following neutrophil migration after 1 or 4 hours. **(C)** Epithelial cell and neutrophil nuclei were counted in ImageJ and the average number of nuclei from all images was calculated. Bars show mean ± SEM of n = 5 biological repeats. Statistical significance is shown. A hash (#) indicates a significant (P < 0.05) difference was found when compared to the equivalent data collected at 24 hours post infection (Fig. 6). For all other comparisons P > 0.05.

Airway epithelial cell damage was further increased (P < 0.05) after 72 hours RSV-infection (Fig. 7) compared to 24 hours (Fig. 6) in relation to TEER etc. After 72 hours neutrophil migration (4 hours) resulted in a significant (P < 0.05) increase in red-dextran leakage and LDH release from RSV-infected epithelial cells compared to cultures mock-infected (Fig. 7A). Neutrophil migration (1 and 4 hours) through AECs infected with RSV for 72 hours also led to a significant (P < 0.05) (1h- 46%, 4h- 41%) reduction in the number of epithelial cells present on the membrane compared to the mock control (Fig. 7B and C). However, neutrophils that migrated in response to fMLP (positive control) did not affect the numbers of epithelial cells present on membranes compared to mock controls, despite reporting similar levels of neutrophil migration as the RSV infected Transwells (see Fig. 3).

## Discussion

We have developed a novel neutrophil and airway epithelial cell (AEC) co-culture system to explore the effect of neutrophil trans-epithelial migration in response to RSV infection. Our study has revealed valuable new insights into the function and activation of neutrophils that could have significant implications for RSV disease development and severity. Previous *in vitro* cell culture models using airway and bronchial epithelial cell lines has provided insight into the mechanisms of RSV infection^13,30,32–35^ but these models have used epithelial cell monolayer in the opposite orientation^33,34^, which does not mimic the correct polarity of trans-epithelial migration of neutrophils *in vivo*. Our inverted epithelial model showed consistent characteristics of RSV infection as traditional epithelial cell models, including RSV replication kinetics, increased inflammatory cytokine production (CXCL8 and IL-6) and epithelial monolayer disruption. Epithelial integrity is a good indication of barrier function and RSV infection is thought to decrease the epithelial monolayer resistance and increase the permeability of polarised AECs^30^. We found increased epithelial cell detachment and increased LDH release after 72 hours RSV infection. This is in agreement with the data performed in other research group using cell lines for RSV infection^30^ and similar to what has been observed in human lungs^36^. Under these conditions, the epithelium is compromised in its defensive functions and therefore 72 hours post RSV infection was chosen as an appropriate end point for this study.

The primary objective of neutrophils is to migrate out of the blood circulation and across a number of tissue barriers and then degranulate, phagocytose and destroy pathogenic microorganisms in the acute inflammatory response^37^. We found that neutrophil trans-epithelial migration was dependent on soluble factors produced by RSV infected AECs. This is in good agreement with previous reports which showed epithelial cells drive active migration through the production of chemoattractants in response to RSV, particularly CXCL8^38–40^, which we showed was increased in our infection model. In order to characterise the function of migrated neutrophils, we measured the expression of two important markers of neutrophil activation, CD11B and myeloperoxidase (MPO). Neutrophil transepithelial migration is thought to be exclusively dependent on CD11B^26,41–43^, which mediates neutrophil interaction with the basolateral and apical epithelial surface^44–47^. Furthermore, neutrophils isolated from BAL of children with severe RSV disease have increased expression of CD11B compared to neutrophils isolated from the blood^31^. In our model, neutrophil expression of CD11B (Fig. 4) correlated with RSV infection and epithelial cell detachment compared to mock controls. MPO expression was also consistently higher in neutrophils that migrated through RSV infected epithelium compared to mock controls at all time points investigated. MPO serves as an index of neutrophil activation and degranulation^48^ and can influence metabolic reactions as well as the amount and type of oxidants production^49,50^. Increased levels of MPO have been reported in viral infections^51^ and RSV-stimulated neutrophils have been shown to contribute to the pulmonary pathology in RSV bronchiolitis^52^.

We also found that, particularly at the early (24 hours) stage of RSV infection, CD11B and MPO expression was higher in neutrophils that had migrated through RSV infected AECs compared to those that remained on the basolateral side (non-migrated neutrophils) (Fig. 5A). This suggests that transepithelial migration is a key factor in stimulating neutrophil activation marker expression in our model. Interestingly, we found that MPO expression was also increased in non-migrated neutrophils in RSV infected wells compared to non-migrated neutrophils in the mock infected controls (Fig. 5B). Non-migrated neutrophils will not be in direct contact with epithelial cells thought to be the target of RSV infection. These data indicates that soluble factors present in the basolateral side of RSV infected epithelial cells may stimulate MPO expression.

Neutrophil migration is thought to occur without inducing any effects on barrier function^53,54^. However *in vitro*, massive neutrophil migration is associated with enhanced epithelial permeability^55,56^. In our model, neutrophil migration in response to RSV enhanced epithelial damage. Our positive control (fMLP) induced massive neutrophil migration, but did not cause disruption in epithelial layer integrity or increase in cell detachment. This suggests that neutrophil-induced epithelial barrier disruption is dependent on the compromised defensive function of epithelial cells induced by RSV infection. This is consistent with clinical findings that the degree of neutrophilic inflammation correlated with high loads of RSV infection, virus-induced lung injury and disease severity in patients with RSV infection^57–61^.

A549 cells remain a popular choice for studying RSV infection^62,63^. However, compared to other lung epithelial cells, their lower TEER, higher paracellular permeability^64^ and reduced tight junction formation^65^, does limit our interpretation of the epithelial integrity following neutrophil migration and RSV infection. Our TEER readings of 150 Ωcm^2^ at confluency are lower than those reported for primary cells (>700 Ωcm^2^) but are higher than those reported by others using A549 cells (<50 Ωcm^2^)^65^. Therefore we are confident that our cell layer is confluent and that the integrity of the A549 barrier is limited only by the reduced presence of tight junction proteins in these cells. We therefore recommend that future studies aimed at determining changes in the epithelial barrier and role of tight junctions during neutrophil migration should use other lung epithelial cells (i.e Calu-3) or primary lung epithelial cell cultures.

In conclusion, we have developed a physiologically relevant co-culture system to study the role of neutrophils in RSV infection. This model will allow us to identify critical mechanisms that mediate epithelial cell damage and promote inflammation in children with severe RSV bronchiolitis.

## supplementary material

Supplementary Dataset 1
