# Supplementary material for: An in vitro transepithelial migration assay to evaluate the role of neutrophils in Respiratory Syncytial Virus (RSV) induced epithelial damage
Source: Sci Rep. 2018 Apr 30;8:6777. doi: 10.1038/s41598-018-25167-4 (PMC5928117; doi:10.1038/s41598-018-25167-4)

**An in vitro transepithelial migration assay to evaluate the role of neutrophils in Respiratory Syncytial Virus (RSV) induced epithelial damage.**

**Yu Deng, Jenny A. Herbert, Claire M. Smith, Rosalind L. Smyth**

**Supplementary figures**

Figure S1 –TEER of A549 monolayers post seeding and post neutrophil migration.

TEER was measured using a voltohmmeter. TEER of A549 cultures was measured post cell seeding on transwells at 0, 24, 48, 72 and 96 hours. Bars show mean ±SEM of at least n=2 biological repeats (A). The damage caused to the A549 epithelium following neutrophil migration through cells infected with mock or RSV infection for 24 and 72 hours was determined by TEER. Bars show mean ±SEM of at least n=5 biological repeats. Statistical significance is shown.


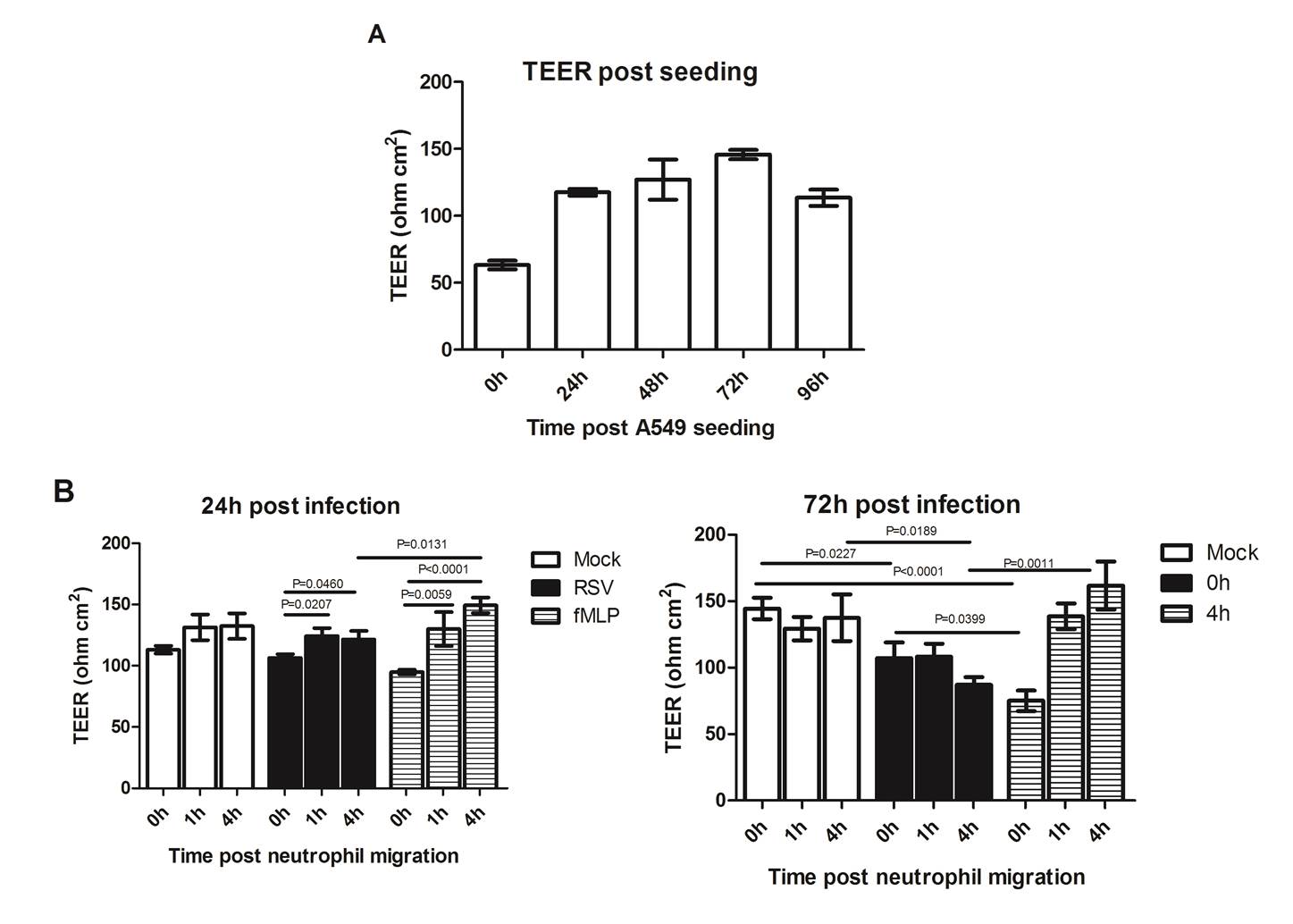


Figure S2 – TEER of A549 monolayers post neutrophil migration

The transepithelial electrical resistance (TEER) of A549 monolayers infected with RSV or mock control post neutrophil migration (0, 1 and 4 hours), as measured using a voltohmmeter. Data is represented as the percentage TEER relative to the mock infected control. Bars show mean ±SEM of n=3 biological repeats. Statistical significance is shown.


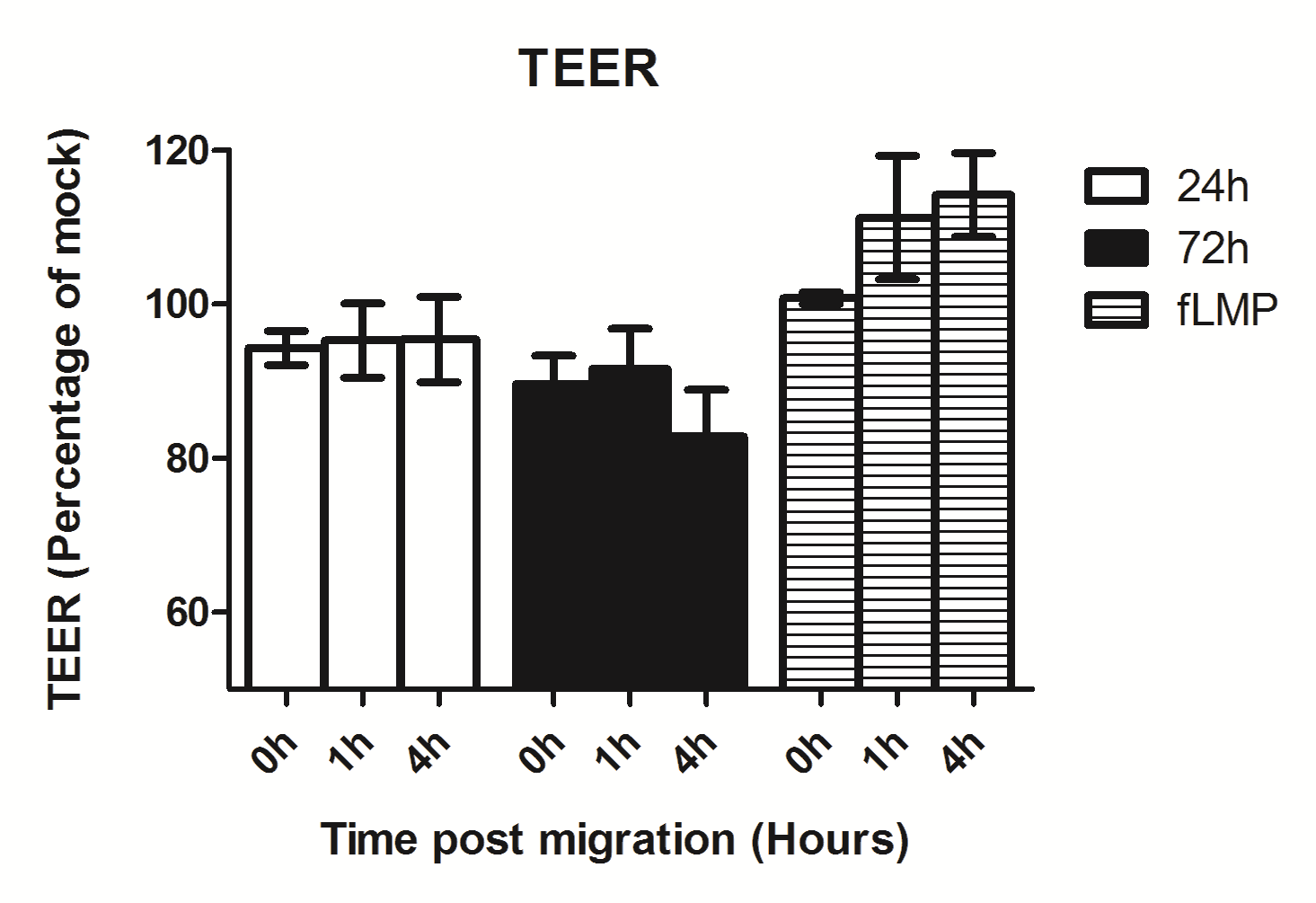


Figure S3 - Number of neutrophils migrated at varying infection time points

Average number of neutrophils that migrated through infected A549 monolayers was quantified at 1 hour and 4 hours through mock and RSV infected monolayers for 24, 48, 72 and 96 hours. Apical fluid was HBSS+ or infection media present under the Transwell prior to migration (supernatant), which contains A549 produced chemokines and cytokines. The average number of neutrophils migrated at each time point was calculated using the percentage migration data in Figure 3 and the total number of neutrophils migrated calculated based on the initial input of 5x10^5^ neutrophils per Transwell.


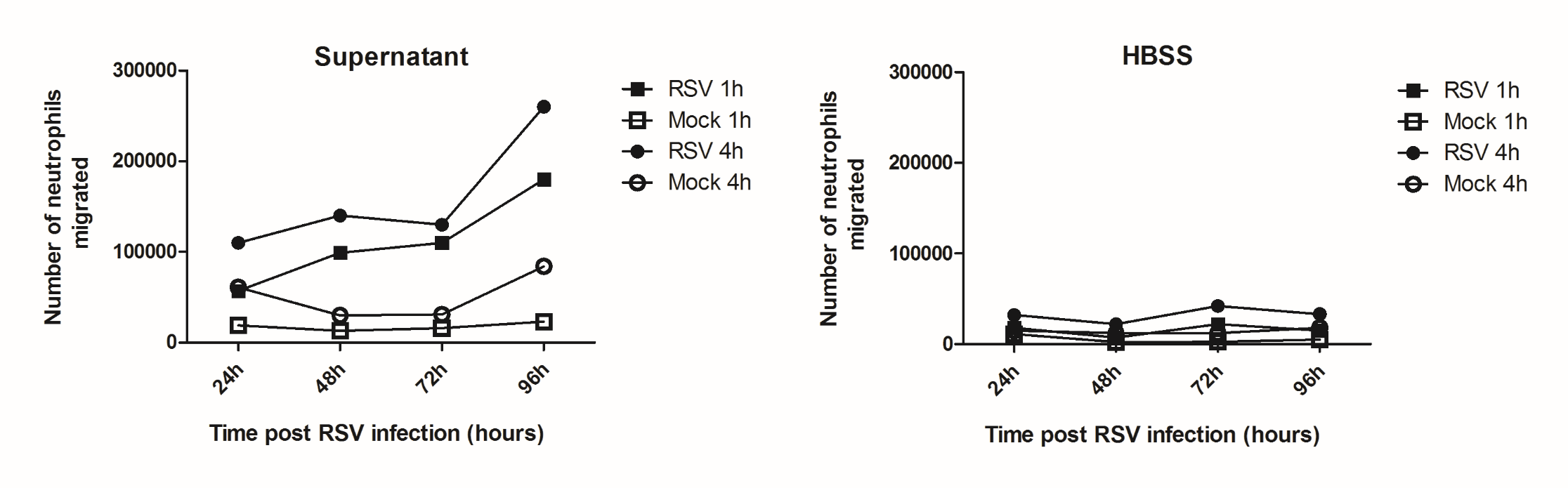


Figure S4 –Numbers of neutrophils that migrate through infected A549 monolayers

The number of neutrophils that migrated through mock and RSV infected A549 monolayers after **(A)** 24 and 72h hours post-infection was quantified. Neutrophils were exposed to infected A549 epithelial cells submerged in fresh HBSS^+^ for 30, 60, 90, 120, 180 and 240 minutes. Graphs show the total number of neutrophils migrated at each time point (A) and the cumulative number of neutrophils migrated over the whole time period (B). n=3 biological repeats.


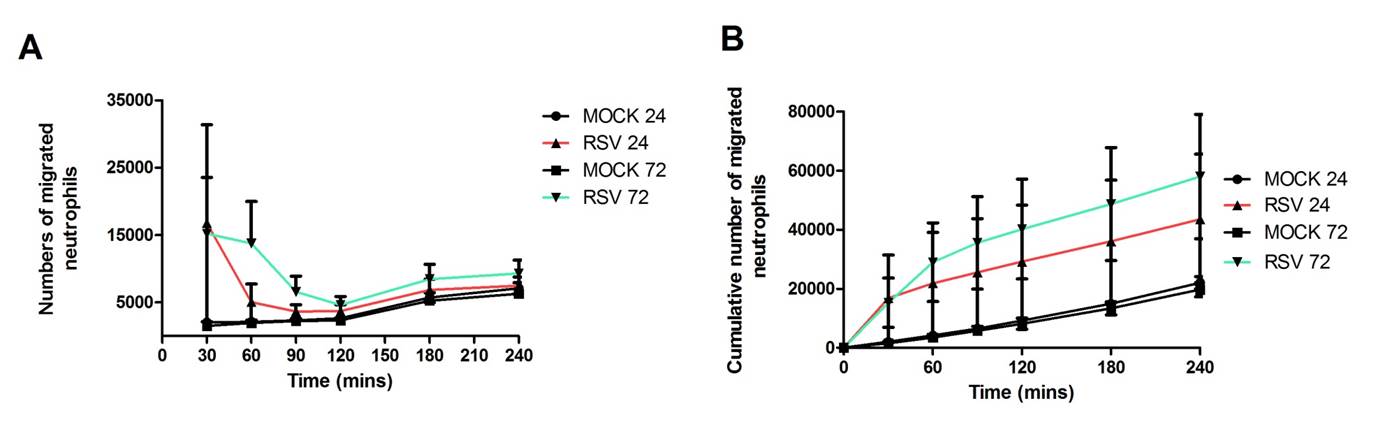

Supplement: Supplementary file 1 — Supplementary Dataset 1 [file 41598_2018_25167_MOESM1_ESM.doc]
